# Colocalized neurotransmitters in the hindbrain cooperate in adaptation to chronic hypernatremia

**DOI:** 10.1007/s00429-020-02049-y

**Published:** 2020-03-21

**Authors:** Rita Matuska, Dóra Zelena, Katalin Könczöl, Rege Sugárka Papp, Máté Durst, Dorina Guba, Bibiana Török, Peter Varnai, Zsuzsanna E. Tóth

**Affiliations:** 1 Department of Physiology, Semmelweis University, Budapest, Hungary; 2 Behavioral Neurobiology, Institute of Experimental Medicine, Budapest, Hungary; 3 Centre for Neuroscience, Szentágothai Research Centre, Institute of Physiology, Medical School, University of Pécs, Pécs, Hungary; 4 Department of Anatomy, Histology and Embryology, Semmelweis University, Budapest, Hungary; 5 Human Brain Tissue Bank and Microdissection Laboratory, Semmelweis University, Budapest, Hungary; 6 Janos Szentagothai School of Neurosciences, Semmelweis University, Budapest, Hungary

**Keywords:** Prolactin-releasing peptide, Noradrenaline, Nesfatin-1, Restraint, Brattleboro rat, Stress

## Abstract

Chronic hypernatremia activates the central osmoregulatory mechanisms and inhibits the function of the hypothalamic–pituitary–adrenal (HPA) axis. Noradrenaline (NE) release into the periventricular anteroventral third ventricle region (AV3V), the supraoptic (SON) and hypothalamic paraventricular nuclei (PVN) from efferents of the caudal ventrolateral (cVLM) and dorsomedial (cDMM) medulla has been shown to be essential for the hypernatremia-evoked responses and for the HPA response to acute restraint. Notably, the medullary NE cell groups highly coexpress prolactin-releasing peptide (PrRP) and nesfatin-1/NUCB2 (nesfatin), therefore, we assumed they contributed to the reactions to chronic hypernatremia. To investigate this, we compared two models: homozygous Brattleboro rats with hereditary diabetes insipidus (DI) and Wistar rats subjected to chronic high salt solution (HS) intake. HS rats had higher plasma osmolality than DI rats. PrRP and nesfatin mRNA levels were higher in both models, in both medullary regions compared to controls. Elevated basal tyrosine hydroxylase (TH) expression and impaired restraint-induced TH, PrRP and nesfatin expression elevations in the cVLM were, however, detected only in HS, but not in DI rats. Simultaneously, only HS rats exhibited classical signs of chronic stress and severely blunted hormonal reactions to acute restraint. Data suggest that HPA axis responsiveness to restraint depends on the type of hypernatremia, and on NE capacity in the cVLM. Additionally, NE and PrRP signalization primarily of medullary origin is increased in the SON, PVN and AV3V in HS rats. This suggests a cooperative action in the adaptation responses and designates the AV3V as a new site for PrRP’s action in hypernatremia.

## Introduction

Homeostatic regulation of plasma osmolality (275–290 mOsm/kg) is vital. Elevated osmolality develops primarily based on elevated extracellular [Na^+^] level known as hypernatremia. Besides activating the central osmoregulatory system, hypernatremia also represents a serious stress for the organism that impairs the response of the hypothalamic–pituitary–adrenal (HPA) axis to a novel acute stressor (Amaya et al. 2001; Krause et al. 2017). The mechanism is still unclarified, therefore we focused our research on brain structures that participate in the control of both hypernatremia and stress-evoked responses, such as the A1 and A2 noradrenaline (NE) cell groups in the caudal ventrolateral (cVLM) and dorsomedial (cDMM) medulla oblongata.

Hypertonic saline administered by intravenous infusion evokes neuronal activation (Fos positivity) in the A1 and A2 NE cells specifically, as infusions of physiological or hypotonic NaCl solutions fail to activate these neurons (Hochstenbach and Ciriello 1995). NE is an essential transmitter for stimulating areas in the hypothalamus that govern the neuroendocrine, autonomic and behavioral responses for hypernatremia (Day 1989; Tanaka et al. 1997; Huang et al. 2000; Bourque 2008; Pedrino et al. 2012; da Silva et al. 2013; Sawchenko and Swanson 1981; Pacak et al. 1995). These areas receive their NE innervation primarily from the neurons of the A1, A2 cell groups and include the paraventricular (PVN) and supraoptic (SON) nuclei as well as the periventricular anteroventral third ventricle region (AV3V). The latter comprises the organum vasculosum laminae terminalis (OVLT), the median preoptic nucleus (MnPO) and the preoptic and anteroventral periventricular nuclei (Bourque 2008; Menani et al. 2014).

The neurons of the A1, A2 cell groups also convey stress-related stimuli that signal homeostatic perturbations (Liposits et al. 1986; Ulrich-Lai and Herman 2009; Gaillet et al. 1991) towards the HPA axis, namely towards the corticotropin-releasing hormone (CRH) producing cells in the parvocellular PVN. The PVN is innervated by NE fibers almost exclusively (94%) from the A1 and A2 cell groups (Sawchenko and Swanson 1981, 1982). Bilateral pharmacological lesions of the ascending medullary NE fibers lead to a 90% decrease of CRH released into hypophyseal portal vessels (Guillaume et al. 1987) and obliteration of the diurnal increment in plasma adrenocorticotropic hormone (ACTH) (Szafarczyk et al. 1985) under basal conditions. Similar lesions caused dramatically reduced ACTH and impaired corticosterone responses to acute ether or restraint stress (Gaillet et al. 1991; Pacak et al. 1995). Chemical ablation of the catecholaminergic terminals within the PVN also attenuated peak ACTH and corticosterone levels in response to acute restraint (Flak et al. 2014).

Despite these facts, the significance of the A1 and A2 cells in adaptation to chronic hypernatremia as well as in altered responsiveness of the HPA axis to acute restraint in hypernatremic animals has not been elucidated yet. Moreover, the A1 and A2 cells very intensely coexpress prolactin-releasing peptide (PrRP) and nesfatin-1/NUCB2 (nesfatin) (Chen et al. 1999; Toth et al. 2008; Konczol et al. 2010) together with NE. Therefore, we assume that cooperation of these substances may be relevant. Indeed, enhanced expression of different cotransmitters is a fundamental event in adaptation to chronic restraint stress (Ma et al. 1997; Amaya et al. 2001; Toth et al. 2008). PrRP cells comprise a large subpopulation of the A2 cells (~ 82%) in the nucleus of the solitary tract (NTS) and practically correspond to all A1 (~ 98%) neurons. They, therefore, coexpress tyrosine hydroxylase (TH, the rate-limiting enzyme of the NE synthesis) and PrRP (Chen et al. 1999; Toth et al. 2008). This feature helps to identify PrRP axons of medullary origin at the terminal fields, as the third PrRP cell population in the brain, which exists in the dorsomedial nucleus of the hypothalamus, is TH negative (Roland et al. 1999). PrRP is involved in various functions challenged by hypernatremia, like the cardiovascular regulation (Yamada et al. 2009) and the control of oxytocin (promotes sodium excretion) and vasopressin (AVP, stimulates water retention) release from the magnocellular cells of the SON and PVN (Maruyama et al. 1999; Uchida et al. 2010). PrRP neurons in the A1 and A2 cell groups are strongly activated (~ 90% Fos positivity) by water immersion-restraint stress, while PrRP-negative A2 neurons barely respond to this stimulus (Maruyama et al. 2001). Acute restraint upregulates PrRP mRNA expression of the A1 cell group (Mera et al. 2006; Konczol et al. 2010). PrRP cells synapse with CRH neurons in the PVN and central administration of PrRP induces Fos protein accumulation in CRH neurons as well as a CRH-mediated increase in the ACTH level (Seal et al. 2002; Maruyama et al. 2001). Although a synergistic effect of PrRP and NE was observed on ACTH (Maruyama et al. 2001) and AVP releases (Uchida et al. 2010), the contribution of PrRP to chronic hyperosmotic responses in the PVN, SON and AV3V has not been investigated yet and the origin of the PrRP fibers at these locations is unrevealed.

Nesfatin-1, the N-terminal fragment of the NUCB2 prohormone is expressed in several brain nuclei, for example, in the PVN and SON (Foo et al. 2008). The vast majority of the nesfatin neurons in the cVLM and cDMM are TH and PrRP coexpressing cells (Konczol et al. 2010). Nesfatin is not transported axonally, but acts locally by autocrine/paracrine fashion (Foo et al. 2008). Nesfatin has been attributed to similar autonomic and endocrine functions to PrRP (Konczol et al. 2010; Maejima et al. 2009; Yilmaz et al. 2015; Yosten and Samson 2009). As these effects were revealed by intracerebroventricular (icv) injections, the exact sites of actions are unknown. We earlier found, however, that acute restraint elevated nesfatin expression in the cVLM indicating its role in the stress response (Konczol et al. 2010).

Based on the above facts, we addressed the following questions: 1, whether the level of the stress caused by chronic hypernatremia may depend on the type of hypernatremia (i.e., eu- or hypervolemic, congenital or acquired); 2, whether the different types of chronic hypernatremia affect the responsiveness to acute restraint differentially; and 3, whether the expression of TH, PrRP and nesfatin in the cDMM and cVLM reflects the actual sensitivity of the HPA axis to acute restraint under chronic hypernatremic conditions. Additionally, we also examined the morphological basis for a putative interaction of NE and PrRP of medullary origin in the hypothalamus (PVN, SON, AV3V) in response to chronic hypernatremia. We used two kinds of models: (1) Brattleboro rats homozygous for diabetes insipidus (euvolemic, DI rats) due to hereditary AVP deficiency and (2) Wistar rats receiving high salt (2% NaCl) solution instead of tap water (hypervolemic, HS rats) for 6 days.

## Methods

### Animals

The subjects of the study were adult (12–14 week old) male Wistar (Toxi-Coop Toxicological Research Center Zrt, Dunakeszi, Hungary) and homozygous Brattleboro rats (+/+ wild type; WT and −/− AVP deficient with diabetes insipidus; DI). The Brattleboro rats were maintained in a colony started from breeder rats from Harlan (Indianapolis, IN), as earlier described (Varga et al. 2016). The rats were housed in a controlled environment (23 ± 1 °C, 50–70% humidity, 12 h light starting at 07:00) and had free access to standard rodent chow and tap water, except otherwise indicated. Due to the extremely high level of urination caused by the diabetic condition, we changed sawdust bedding daily for all rats. Animals were anesthetized with ketamine (75 mg/bwkg) (Richter Gedeon Nyrt., Budapest, Hungary) and xylasine (15 mg/bwkg) (CP-Pharma, Burgdorf, Germany) given intramuscularly prior to perfusion fixation.

### Experimental design

#### Experiment 1

The Brattleboro rats were divided into four groups: WT-control, DI-control, WT-restraint (R) and DI-R (*n* = 7/group). Bodyweight, food and water intake of rats were recorded daily in the morning for 5 consecutive days before the experiment started.

The Wistar rats were also assigned into four experimental groups: normal salt (NS)-control, high salt (HS)-control, NS-R and HS-R (*n* = 8/group). Animals in the HS groups received 2% NaCl solution instead of tap water for 6 days. Bodyweight, daily food and water intake of rats were measured on every 3rd day (starting on day zero) to minimize extra stress.

On the day of the experiments, all animals in the R groups were subjected to a single restraint stress as described below. Control subjects remained in their home cages. Blood from the tail vein was collected for hormonal measurements 1 h after restraint had started both from stressed and non-stressed animals. At the end of the restraint, all animals were sacrificed by decapitation. The thymi and adrenal glands were dissected out and their weights were measured. The brains were removed, frozen on dry ice and used for in situ hybridization histochemistry.

#### Experiment 2

The Wistar rats were assigned to two experimental groups, NS and HS (*n* = 8/group). Animals in the HS group received 2% NaCl solution instead of tap water for 6 days. At the end, animals were sacrificed either by decapitation (*n* = 4/group) or by transcardial perfusion with 4% paraformaldehyde in 0.1 M phosphate-buffered saline (PBS, pH = 7.4) (*n* = 4/group). The brains were removed and processed for western blot (fresh-frozen, *n* = 4/group) or immunohistochemistry (perfused-fixed, *n* = 4/group).

### Restraint stress

Single, long-duration restraint was performed as previously described (Zelena et al. 2003). The animals were placed into transparent plastic tubes (5–6 cm inner diameter) having a 4-cm-long conical head part ending with a large breathing hole. Behind the body, the rear end of the tube was loosely packed with paper towels. This method allows restraint without any pain and minimizes temperature effects. Restraint lasted for 3 h in the morning (08:00–11:00) and was finished by transcardial perfusion or by decapitation of the animals.

### In situ hybridization

Serial coronal sections (12 μm thick, 120 μm apart) of the brains were cut using a cryostat (Leica Microsystems, Nussloch, Germany) and mounted on Superfrost Ultra Plus slides (ThermoFisher Scientific, Budapest, Hungary). The slides were stored at − 80 °C until used.

Alternate series of sections from the caudal medulla oblongata were hybridized for either PrRP, TH or NUCB2. One series of hypothalamic sections was hybridized for PrRP receptor (PrRP-R alias GPR10). The S35-UTP-labeled (PerForm Hungaria Kft., Budapest, Hungary) riboprobes were prepared by in vitro transcription as described previously (Konczol et al. 2010; Toth et al. 2008). The specific sequences used for the hybridizations were: rat prepro-PrRP cDNA (10–240 bp of the coding sequence, accession # AB015418), rat TH cDNA (684–1068 bp of the coding sequence, accession # M23598) (Mezey et al. 1998), rat NUCB2 cDNA corresponding to the nesfatin-1 fragment (1–246 bp of the coding sequence, accession # DY314804) and the rat PrRP-R cDNA (189–636 bp of the coding sequence, accession # NM_139193) (Vas et al. 2013; Durst et al. 2019). The specificity of the cDNAs was verified by sequencing. Specificity of the probes was also controlled by performing experiments using sense probes failing to give any signals. After hybridizations, sections were apposed to BAS-MS imaging plates (Fuji Photo Film Co., LTD., Kanagawa, Japan, NJ) for 3 (PrRP, TH) or 7 (NUCB2) days. Data were read out by a Fujifilm FLA-8000 Image Analyzer (Fujifilm Life Science, Stamford, CT). NUCB2 as well as PrRP-R labeled sections were dipped in Kodak NTB nuclear emulsion (Carestream Health Inc., Rochester, NY) for 2 and 6 weeks, respectively, according to the manufacturer’s instructions and developed using Kodak developer and fixer (Sigma). The sections were stained with Giemsa and coverslipped using DPX (Sigma).

### Quantitative analysis of the in situ hybridization signals

Radioactive in situ hybridization provides a linear relationship between the signal intensity and the mRNA expression level (Chen et al. 2012). The signals in the cVLM and cDMM (i.e. NTS) were evaluated on parallel series of sections, between 14.04 and 14.70 mm caudal from bregma (Paxinos and Watson 2007) corresponding to the most sensitive areas for restraint stress (Dayas et al. 2001). The rostro-caudal level of the sections was carefully matched between the animals. Optical densities (mean grey values) of the signals were measured in the phosphor imager recordings in at least four sections/animal using the same settings across animals. Background values measured in parallel were subtracted. NUCB2 signal in the cVLM was weak in the screen (see the Results), therefore, it was measured in darkfield images (Olympus BX60, UPlan FL 4x/0.13) made of the autoradiographic emulsion-coated sections (SPOT Xplorer 17.4 camera, Diagnostic Instruments Inc., Sterling Heights, MI). The amount of the silver grains over the individual cells was evaluated within identical ROIs and expressed as the area (pixels) covered by grains (Wittmann et al. 2015). The signal threshold for each cell was calculated as the mean pixel value of 2–3 background samples measured in the surrounding region. Only cells with pixel values above these criteria were included in the analysis. In all cases, the sections were evaluated bilaterally with the help of the ImageJ program (Wayne Rasband NIH, Bethesda, MD), as earlier described (Vas et al. 2013; Toth et al. 2008). The average/animal data were compared statistically. All measurements were made by an experimenter blind to the experimental settings.

### Hormone measurements

Blood was collected in K-EDTA containing tubes on ice. After centrifugation, the plasma was stored at − 20 °C. ACTH and corticosterone levels were measured from unextracted plasma (50 and 10 μl, respectively) in duplicates with a radioimmunoassay, as described earlier (Zelena et al. 2003). The intra-assay coefficient of variation was 12.5% and 17.6% for ACTH and corticosterone measurements, respectively. All samples from one experiment were measured in one assay.

### Microdissection of selected brain nuclei

Fresh frozen brains were cut into 200-μm-thick slices in a cryostat (Leica), mounted on pre-cooled, untreated glass slides (ThermoFisher) and stored at − 80 °C until used. The regions of the cVLM and cDMM, the SON, the PVN and the AV3V were dissected out guided by the Rat Brain Atlas (Paxinos and Watson 2007), using a modified Palkovits punch technique (Palkovits 1973). Briefly, for dissection, the slides were placed back into the cryostat and kept there during the whole procedure at – 10 °C. The individual nuclei were visualized using a head magnifier and removed from the sections using sterile punch needles with an inside diameter of 0.7 mm. The punch samples were collected immediately in ice cold Eppendorf tubes containing 100 µl of radioimmunoprecipitation assay buffer (RIPA, 50 mM TrisHCl, 150 mM NaCl, 1 mM EDTA, 1% Triton X-100, 0.1% SDS, 0.25% Na-deoxycholate, pH = 7.4) and a cocktail of protease and phosphatase inhibitors (ThermoFisher). Samples were homogenized, lysed and then stored at − 80 °C until used for western blot.

### Western blot

Samples were centrifuged to separate cell debris and nuclei (20,000 *g*, 30 min at 4 °C). The protein concentrations in the supernatant were measured using the Pierce™ BCA Protein Assay Kit (ThermoFisher) according to the instructions of the manufacturer. Samples containing identical brain areas were diluted to equal final protein concentrations and denatured in 2 × Laemmli buffer (Bio-Rad Magyarország Kft., Budapest, Hungary). Proteins (10 μg/sample) were separated in a 13.5% SDS-PAGE gel with 10% stacking gel. After transferring onto PDVF membranes (Merck Millipore, Budapest, Hungary), the membrane-bound protein samples were blocked in 5% BSA for 1 h and subsequently exposed to one of the following primary antibodies: anti-nesfatin-1 (1–82) (Phoenix Europe GmbH, Karlsruhe, Germany, CAT# H-003–22, dilution 1:2000), anti-TH (Merck, CAT# AB152, dilution 1:1000), anti-TH phosphorylated at Ser31 (Sigma, CAT# SAB4300674, dilution 1:1000), anti-PrRP-R (ThermoFisher, CAT# PAS 75,372, dilution 1:1000), all made in rabbit, and then to anti-β-actin (Sigma, CAT# A1978, made in mouse, dilution 1:5000). Signals were visualized using HRP-conjugated goat anti-rabbit or mouse antibodies (Cell Signaling Technology Europe B.V., Frankfurt, Germany, dilution 1:5000) and Chemiluminescent HRP Substrate (Merck), or by fluorescently labeled goat anti-mouse IgG (AzureSpectra 700, Azure Biosystems, Dublin, CA, dilution 1:1000) and detected with the Azure c600 imaging system (Azure Biosystems). Densitometric analyses of the protein bands were performed with the help of the ImageJ application. The average/animal data (*n* = 4) normalized to β-actin were compared statistically.

### Immunohistochemistry

Serial coronal free-floating sections (50 μm-thick, 200 μm apart) from the hindbrain and from the hypothalamus were immunostained for PrRP. One series of hypothalamic sections from a control Wistar rat was double immunostained for PrRP and TH. The sections were incubated in 1% BSA containing 0.5% TX-100 (Sigma) in PBS for 1 h, to block non-specific binding sites in the tissues and to enhance penetration of the antibodies. The sections were then boiled using a microwave in 0.1 M citric acid (pH = 6.0), to perform antigen retrieval and eliminate endogenous HRP activity simultaneously (Toth and Mezey 2007). The rabbit anti-PrRP antibody (https://www.phoenixpeptide.com/products/view/Antibodies/H-008-52, Phoenix, dilution 1:8000) was applied for 2 days at 4 °C. Visualization was performed using the Vectastain-ABC-HRP Kit (Vector Laboratories, Burlingame, CA, CAT# PK 6100) and FITC-tyramide reaction (PerForm, CAT# NEL701A001KT). Double immunostained sections were then further incubated in mouse anti-TH (Millipore, Temecula CA, CAT# MAB318, https://www.merckmillipore.com, dilution 1:200) for 2 days at 4 °C, and in donkey anti-mouse AlexaFluor647 (ThermoFisher, dilution 1:100) for 2 h at room temperature. Control immunostainings were performed by omitting the primary antibodies from the reactions and showed no signals. The sections were mounted on precleaned slides and coverslipped with Vectashield mounting medium (Vector).

### Evaluation of the PrRP immunostained sections

Hindbrain sections immunostained for PrRP were evaluated between 14.04 and 14.70 mm caudal from the bregma level. Hypothalamic sections were analyzed between the − 0.12 and + 0.24 mm, the 0.6–1.08 mm and the 1.3–1.8 mm relative levels to bregma, and contained the AV3V, the SON and the PVN, respectively (Paxinos and Watson 2007). Sections were scanned on a confocal laser scanning system using the same settings across the animals (Zeiss, LSM780, Plan-Apochromat 10×/0.45 M27, pixel size: 0.8303 µm^2^, optical thickness: 25 µm). The number of PrRP-positive cells was counted in the cVLM and NTS in at least two sections per animal bilaterally, using the touch-count tool in the ImageJ application. The density of the immunoreactive fibers was determined in regions of interest (ROI: MnPO: 260 µm × 280 µm, PVN: 200 µm × 200 µm, SON: 40 µm × 40 µm) in two–three sections per animal, bilaterally. The area of the fibers within each ROI was determined with the help of the threshold function in the ImageJ application and divided with the area of the ROI. Measurements were made by an experimenter blind to the experimental settings. The average/animal data (*n* = 4) were compared statistically.

### PrRP and TH coexpression analysis

Coexpression was measured in the above-mentioned hypothalamic areas in high magnification (Plan-Apochromat 60×/1.4 VC) confocal images (Bio‐Rad Laboratories, Hemel Hempstead, UK). For each field 16–20 single optical sections were acquired at a z‐separation of 2 μm (z‐stack) from two sections/area bilaterally. Images with the most intense signals (at least 6/z-stack) were selected for analyses with the help of ImageJ application. Percentage data are expressed as means ± SEM obtained from the four z-stacks in each areas.

### Statistics

Data analyses were performed by investigators blind to treatments. Statistical significances were calculated by employing the Sigmastat 3.5 application (Systat Software Inc., Chicago, IL). Student’s *t* test (two-tailed) was used when comparing two groups with normal distribution of the data, otherwise Mann–Whitney *U* test was applied. Two-way ANOVA followed by Student–Newman–Keuls post hoc analysis was used for calculating statistical significance for four groups with two treatments. One-way ANOVA with repeated measures was used to analyze fluid and food intake values across the 6 days of HS or NS. Data were assessed for normality and equal variances before running ANOVA analyses. Results are expressed as means ± SEM values. Pearson method was used to calculate correlations. Differences between groups were considered statistically significant when *p* < 0.05.

## Results

### Body parameters, food and water intake

Lower bodyweight (*p* < 0.05) and higher plasma osmolalities (*p* < 0.001) were measured in DI animals compared to the age-matched WT peers. Despite the chronic hypernatremia, the relative adrenal and thymus weights were normal. DI rats consumed less food than WT rats (*p* < 0.01), but since they consumed according to their bodyweight, no alteration in the relative daily food intake was seen. The excessive absolute and relative daily water intake (*p* < 0.001 for both parameters) however, confirmed the diagnosis of diabetes insipidus (Table 1).

**Table 1:** Body parameters, daily food and water intakes of homozygous (+/+ and −/−) Brattleboro rats

	WT	DI
Body weight (g)	408 ± 93	56* ± 15
Plasma osmolality (mOsm/L)	305 ± 43	35*** ± 2
Relative adrenal weight (g/bwkg)	97 ± 59	5 ± 9
Relative thymus weight (g/bwkg)	923 ± 82	815 ± 78
Food intake (g)	24 ± 1	21** ± 1
Relative food intake (g/bwkg)	66 ± 3	63 ± 3
Water intake (g)	31 ± 1	147*** ± 6
Relative water intake (g/bwkg)	76 ± 3	415*** ± 16
*n*	7	7

Food and water consumptions were measured for 5 consecutive days

Means ± SEM, Student’s *t* tests, p* < 0.05, p** < 0.01 and p*** < 0.001 vs. WT

*DI* vasopressin-deficient genotype with diabetes insipidus, *WT* wild-type animals of the Brattleboro strain

**Fig. 1:**
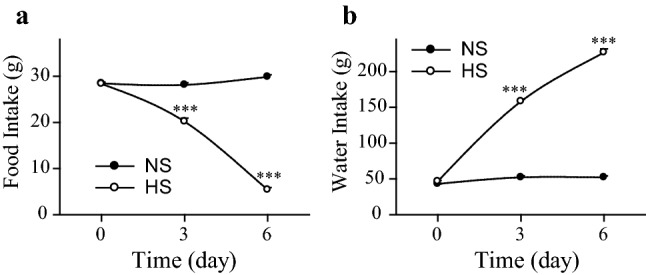
Food (**a**) and fluid (**b**) consumptions of high salt loaded and normal Wistar rats. High salt solution intake evoked dramatic reduction and elevation in food and fluid consumption, respectively. Means ± SEM, Student’s *t* tests, p*** < 0.001 vs. the same day values of non-salt loaded control rats, *n* = 16. *HS* 2% NaCl intake for 6 days, *NS* normal salt solution (tap water) drinking controls

In Wistar rats, high salt intake for 6 days resulted in dramatic weight loss as opposed to weight gain observed in controls (*p* < 0.001). The plasma osmolality was elevated compared to non-challenged (NS) mates (*p* < 0.001), or even DI rats (*p* < 0.01). HS rats suffered from chronic stress; they developed adrenal hypertrophy (*p* < 0.05) and extensive thymus involution (*p* < 0.001) during the treatment (Table 2). Food intake decreased (*p* < 0.001) (Fig. 1a), while fluid intake increased (*p* < 0.001) (Fig. 1b) gradually during HS intake.

**Table 2:** Effect of 2% NaCl solution (high salt; HS) intake for 6 days on body parameters of Wistar rats

	NS	HS
Bodyweight change (g)	49 ± 2	− 46*** ± 5
Plasma osmolality (mOsm/L)	304 ± 1	363*** ± 7
Relative adrenal weight (g/bwkg)	166 ± 8	219* ± 17
Relative thymus weight (g/bwkg)	2349 ± 139	944*** ± 102
*n*	8	8

Control subjects received tap water (normal salt; NS). Means ± SEM, Student’s *t* tests, p* < 0.05 and p*** < 0.001 vs. NS

### Plasma ACTH and corticosterone concentrations

**Fig. 2:**
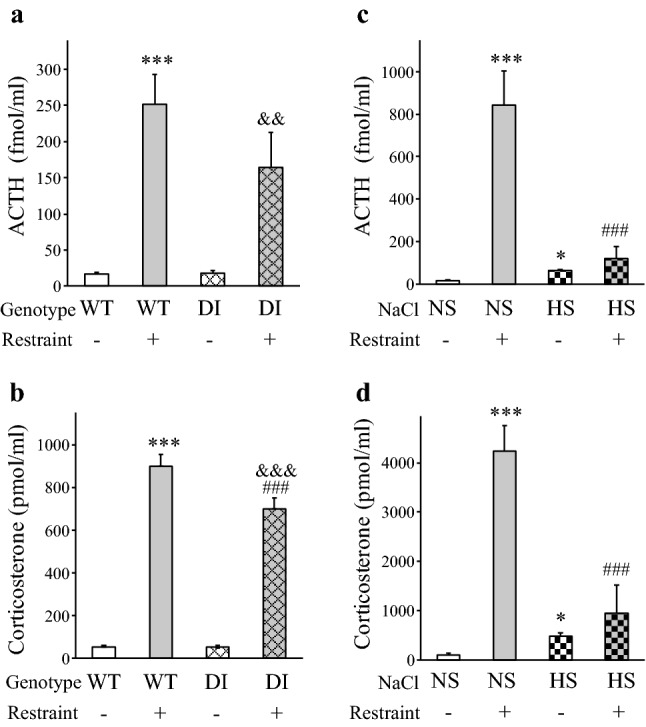
Plasma adrenocorticotropic hormone and corticosterone concentrations in chronic hypernatremia and 1 h after acute restraint stress. ACTH (**a**) and corticosterone (**b**) levels in homozygous WT and DI Brattleboro rats. The basal hormone levels were similar between the WT and DI genotypes. The acute restraint (R) evoked large ACTH and corticosterone elevations in both genotypes, although the corticosterone response in the DI-R group was slightly reduced compared to the WT-R group. ACTH (**c**) and corticosterone (**d**) levels in the HS model. Chronic hypernatremia elicited by HS intake resulted in mild, but significant elevations in basal hormone levels. The acute restraint evoked large ACTH and corticosterone responses in the NS-R group. Hormonal responses were greatly inhibited in the HS-R group. Means ± SEM, Student’s *t* test, p* ≤ 0.001 HS vs. NS control group, Two-way ANOVA, significance indicate the results of Student–Newman–Keuls post hoc tests, p*** ≤ 0.001 vs. the WT or NS control groups, *p*^###^ ≤ 0.001 vs. the WT-R or NS-R group, *p*^&&&^ ≤ 0.001 and *p*^&&^ ≤ 0.01 vs. the DI control group, DI model: *n* = 7; HS model: *n* = 7–8. *ACTH* adrenocorticotropic hormone, *DI* vasopressin-deficient genotype with diabetes insipidus, *WT* homozygous wild-type animals of the Brattleboro strain

The basal ACTH and corticosterone levels were normal in DI rats suggesting the HPA axis was adapted to the congenital hypernatremia (Fig. 2a, b). The acute stress (restraint) evoked large ACTH (WT vs. WT-R: *p* < 0.001, DI vs DI-R: *p* < 0.01) and corticosterone (WT vs. WT-R and DI vs DI-R: *p* < 0.001) release. The corticosterone response was slightly subnormal in DI-R rats (WT-R vs. DI-R: *p* = 0.001) (Fig. 2a, b).

HS intake resulted in elevated basal ACTH and corticosterone levels (Student’s *t* tests, *p* ≤ 0.001 for both parameters) (Fig. 2c, d). The acute restraint evoked large ACTH and corticosterone release (NS vs. NS-R: *p* < 0.001 for both hormones), but the responsiveness of the HPA axis was greatly inhibited in the HS-R group (NS-R vs. HS-R: *p* < 0.001 for both hormones, HS vs. HS-R: no significant differences) (Fig. 2c, d).

Very high correlations were found between the ACTH and corticosterone values in both models (*R*_DI_ model = 0.827, *n* = 28; and *R*_HS_ model = 0.907, *n* = 31, *p* < 0.00001), indicating that the sensitivity of adrenal corticosterone secretion to plasma ACTH levels was maintained.

### TH, PrRP and NUCB2 mRNA levels in the cVLM

**Fig. 3:**
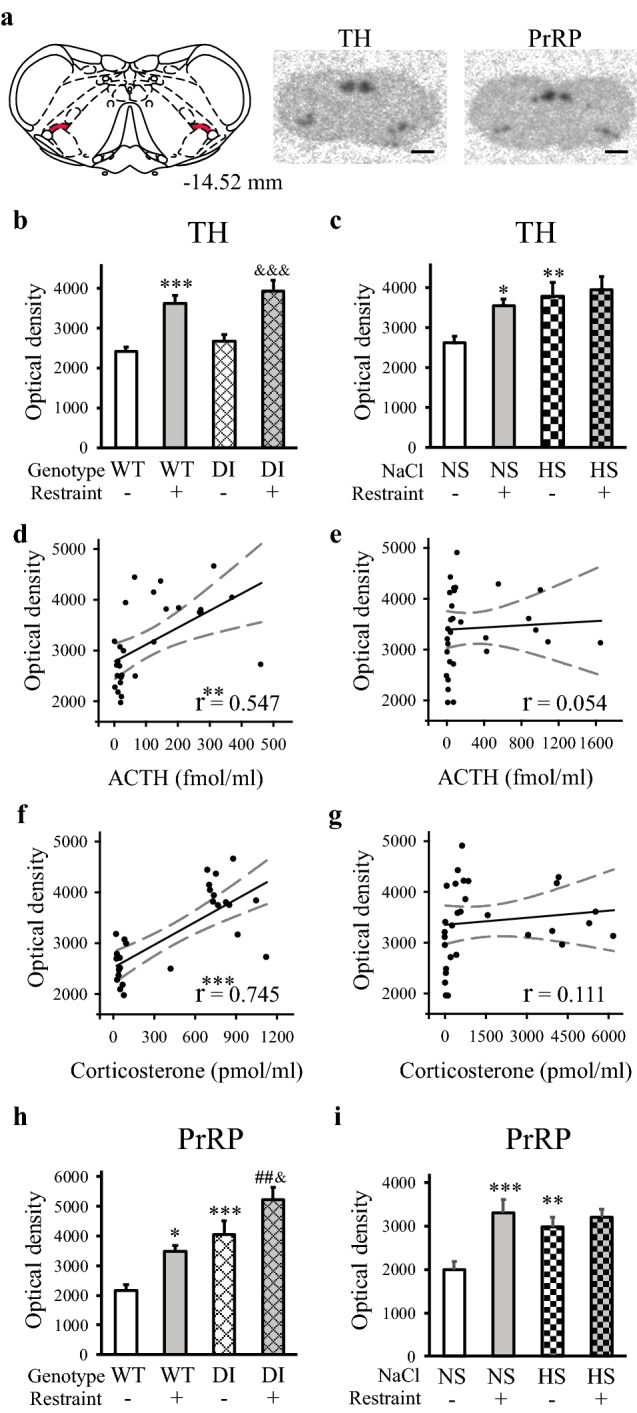
Tyrosine hydroxylase and prolactin-releasing peptide mRNA levels in chronic hypernatremia and 3 h after acute restraint stress in the caudal ventrolateral medulla. **a** Location of the cVLM (left) and illustrative autoradiographic pictures (right). The red shaded regions in the drawing (Paxinos and Watson 2007) label the cVLM. The images display the in situ hybridization signals (dark spots) and were captured by storage phosphor screens. Distance from the bregma is indicated in millimeters. Scale: 4 mm. **b**, **c** TH mRNA. In Brattleboro rats, restraint evoked large increase, whereas the genotype had no effect (**b**). HS or restraint applied separately evoked TH mRNA level elevations in Wistar rats. Prior HS blunted the TH response for restraint (**c**). **d–g** Associations between the TH mRNA and ACTH or corticosterone levels. Both ACTH (**d**) and corticosterone (**f**) values showed strong relationships with TH mRNA levels in the DI model. There were no associations between these data (**e** and **g**, respectively) in the HS intake model. **h**, **i** PrRP mRNA. Both the DI genotype and restraint increased PrRP mRNA levels in Brattleboro rats, resulting in the largest reaction in the DI-R group (**h**). HS or restraint applied separately evoked PrRP mRNA level elevations in Wistar rats. Prior HS blunted the response for restraint (**i**). Bar graphs: means ± SEM, Two-way ANOVA, significance indicate the results of Student–Newman–Keuls post hoc tests, p*** < 0.001, p** < 0.01, or p* < 0.05 vs. WT or NS control groups, *p*^##^ < 0.01 vs. WT-R animals, *p*^&&&^ < 0.001 and *p*^&^ < 0.05 vs. DI control rats, DI model: *n* = 7; HS model: *n* = 8. Correlation graphs: solid and dashed lines show linear regressions and 95% confidence intervals, respectively, see Pearson correlation coefficients (*r*) in the graphs, p*** < 0.001, p** < 0.01, DI model: *n* = 28; HS model: *n* = 31. *PrRP* prolactin-releasing peptide, *TH* tyrosine hydroxylase, *cVLM* caudal ventrolateral medulla

In the cVLM (Fig. 3a), the basal amounts of TH mRNA were similar in the WT and DI rats (Fig. 3b), but differed between the NS and HS animals (NS vs. HS: *p* < 0.01) (Fig. 3c). The acute restraint evoked large increase in TH mRNA levels in the wild-type and DI Brattleboro rats (WT vs. WT-R and DI vs. DI-R: *p* < 0.001) (Fig. 3b), and in the normal Wistar rats (NS vs. NS-R: *p* < 0.05) (Fig. 3c). HS animals with elevated TH mRNA levels however, failed to react to restraint with TH mRNA increase (Fig. 3c). Therefore, TH mRNA levels correlated well with ACTH (*p* < 0.01) and corticosterone (*p* < 0.001) levels in the DI (Fig. 3d, f), but not in the HS intake model (Fig. 3e, g).

PrRP mRNA quantity was heightened by both DI (WT vs. DI: *p* < 0.001) and restraint (WT vs WT-R: *p* < 0.05), resulting in the highest mean value in the DI-R group (WT-R vs. DI.-R: *p* < 0.01, DI vs DI-R: *p* < 0.05) (Fig. 3h). PrRP mRNA changes in the HS model were similar to that of TH mRNA (NS vs. HS: *p* < 0.01 and NS vs. NS-R: *p* < 0.001). The PrRP mRNA response to the acute restraint was entirely impeded in the HS-R animals (Fig. 3i).

**Fig. 4:**
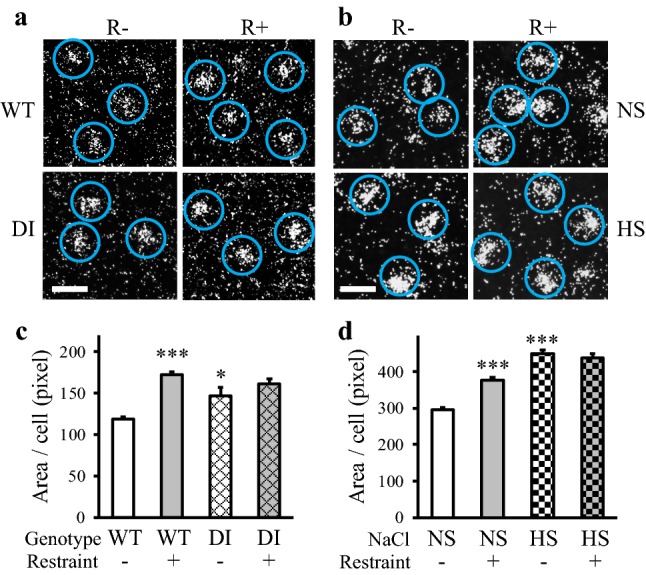
NUCB2 mRNA levels in chronic hypernatremia and 3 h after acute restraint stress in the cVLM. **a**, **b** Illustrative darkfield microphotographs of the autoradiographic emulsion-coated sections displaying NUCB2 mRNA expressing cells encircled by blue ROIs. The in situ hybridization signals are seen as clusters of silver grains (white dots) over the cells. Scales: 40 µm **c**, **d** Chronic hypernatremia and restraint elevated NUCB2 mRNA levels in both models, when applied separately. No reactions were observed in the double challenged rats. Means ± SEM, two-way ANOVA, significance indicate the results of Student–Newman–Keuls post hoc tests, p*** < 0.001, p* < 0.05 vs. WT or NS control rats, DI model: *n* = 4–5; HS model: *n* = 8

NUCB2 mRNA expressing cells were clearly visible in the emulsion-coated sections (Fig. 4a, b). The signal intensities were higher in hypernatremic animals (WT vs. DI: *p* < 0.05, NS vs. HS: *p* < 0.001), and in the restraint stressed groups (WT vs. WT-R and NS vs. NS-R: *p* < 0.001) compared to normal controls. Prior chronic osmotic stress inhibited the NUCB2 responses to restraint in both models (Fig. 4c, d).

### TH, PrRP and NUCB2 mRNA levels in the cDMM

**Fig. 5:**
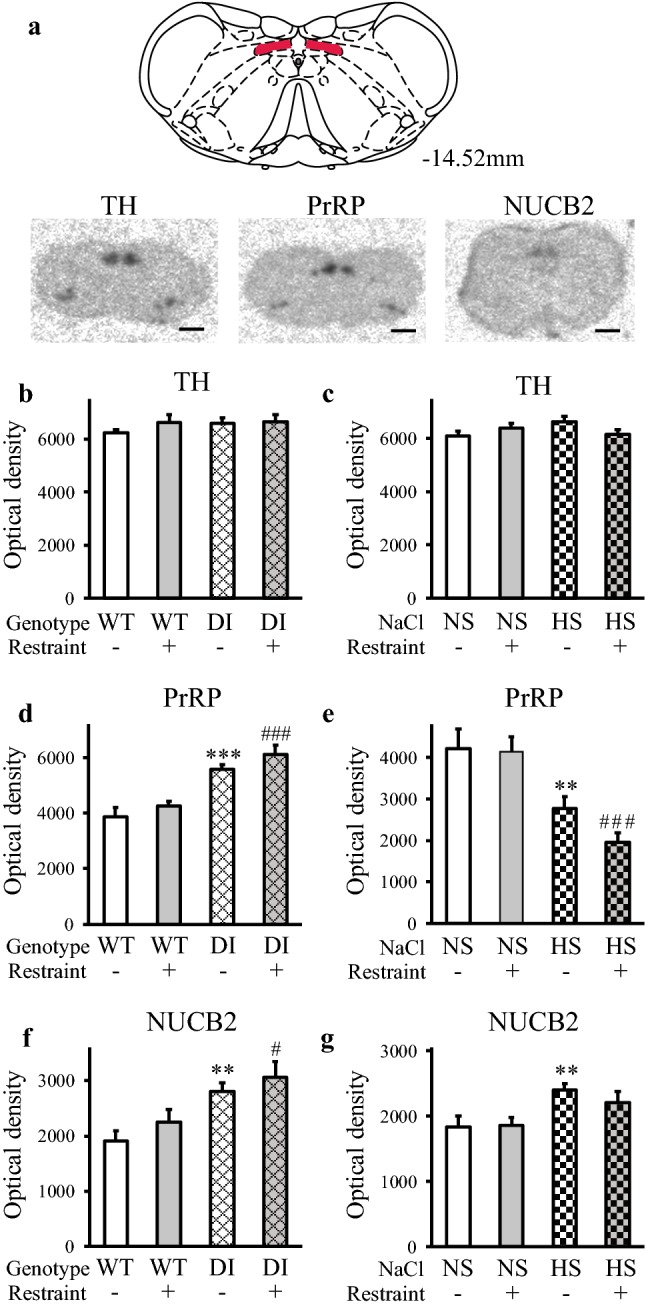
TH, PrRP and NUCB2 mRNA levels in chronic hypernatremia and 3 h after acute restraint stress in the caudal dorsomedial medulla. **a** Location of the measured area within the cDMM (top) and illustrative autoradiographic pictures (bottom). The red shaded regions in the drawing (Paxinos and Watson 2007) label the measured area (nucleus of the solitary tract). The images display the in situ hybridization signals (dark spots), and were captured by storage phosphor screens. Distance from the bregma is indicated in mm. Scale: 4 mm. **b, c** TH mRNA. There were no alterations in either of the hypernatremia models; DI (**b**), HS intake (**c**). **d**, **e** PrRP mRNA. Increased levels were measured in the AVP deficient Brattleboro rats, independently of restraint (**d**). Depressed amounts of PrRP mRNA were detected in Wistar rats challenged by HS intake (**e**). Restraint had no effect in either of the models. **f**, **g** NUCB2 mRNA. Chronic hypernatremia elevated NUCB2 mRNA levels in both models. Restraint had no effect in either of the models. Means ± SEM, Two-way ANOVA, significance indicate the results of Student–Newman–Keuls post hoc tests, p*** < 0.001 and p** ≤ 0.01 vs. WT or NS control rats, *p*^###^ < 0.001 and *p*^#^ < 0.05 vs. WT-R or NS-R animals, DI model: *n* = 7 (**b**, **d**), *n* = 4–5 (**f**); HS model: *n* = 8. *cDMM*: caudal dorsomedial medulla

In the cDMM (Fig. 5a), TH, PrRP and NUCB2 mRNA levels were unaffected by acute restraint in both models (Fig. 5b–g). The amounts of TH mRNA were also normal in the hyperosmotic DI and HS rats (Fig. 5b, c).

PrRP mRNA values were increased in the DI rats (WT vs. DI and WT-R vs. DI-R: *p* < 0.001), (Fig. 5d), but they were reduced by HS intake (NS vs. HS: *p* < 0.01, NS-R vs. HS-R: *p* < 0.001) (Fig. 5e).

NUCB2 mRNA levels have changed similarly to that of PrRP mRNA in the Brattleboro rats and they were elevated by hyperosmolality (WT vs. DI: *p* = 0.01, WT-R vs. DI-R: *p* < 0.05) (Fig. 5f). HS intake also increased the levels of NUCB2 mRNA (WT vs. HS: *p* < 0.01, NS-R vs. HS-R: *p* = 0.085) (Fig. 5g).

### Chronic high salt intake evoked changes in protein expressions in the medulla and the hypothalamus

**Fig. 6:**
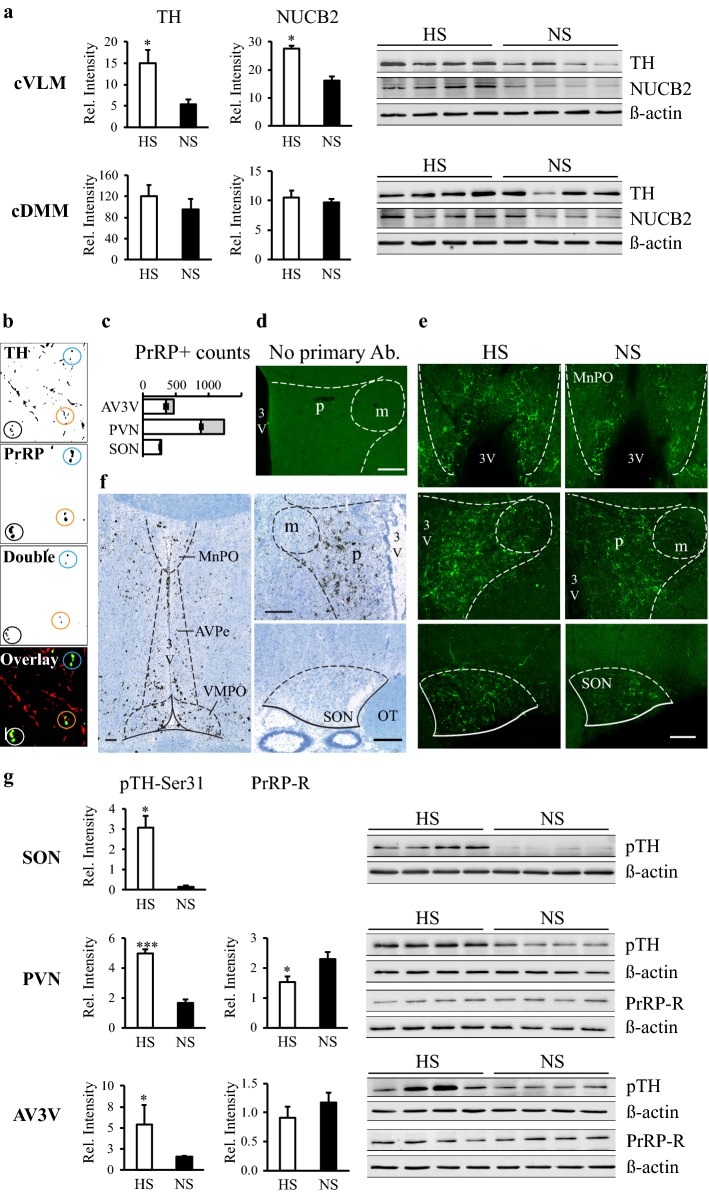
Chronic high salt intake induced changes in protein expressions. **a** Western blots and quantification of TH and NUCB2 signals in the cVLM and cDMM. Intensity values were normalized to β-actin. TH and NUCB2 protein levels were heightened in the cVLM, but not in the cDMM in response to HS intake. **b** Coexpression analysis of TH- and PrRP- immunoreactive profiles in normal Wistar rats. Illustrative, binary, confocal images (single optical layer) and the color-coded overlay picture (red: TH, green: PrRP, yellow: coexpression) of the same field within the SON. The identical circles feature identical elements. Double-labeled elements were calculated by the ImageJ program. Scale: 10 µm. **c** Quantitative results of the coexpression analysis. The number of PrRP-positive profiles were the highest in the PVN. Most of the counted profiles were double-labeled for TH and PrRP (white columns). The amount of the single PrRP-positive profiles were less than 30% in the AV3V and the PVN, and less than 10% in the SON (grey columns). **d**, **e** HS intake elicited changes in PrRP immunoreactivity. Control immunostaining for PrRP in the PVN (**d**). No signal is seen by omitting the primary antibody from the reaction. HS intake increased the density of PrRP-positive fibers in all investigated regions (**e**): the AV3V (MnPO, top), PVN (middle) and SON (bottom). Note, that PrRP fibers expand both in the parvo- and the magnocellular parts of the PVN during HS intake. Scale: 100 µm. **f** Pattern of the PrRP-receptor mRNA expression in the investigated hypothalamic areas. In situ hybridization signals (black silver grain clusters) in the AV3V region (left), the PVN (top right) and the SON (bottom right). Positive cells are present in the AV3V and the parvocellular PVN. Note, signals avoid the magnocellular PVN and the SON. The sections were counterstained with Giemsa. Scales: 100 µm. **g** Western blots and quantification of pTH-Ser31 and PrRP-receptor peptide levels in the hypothalamus. Intensity values were normalized to β-actin. The amounts of pTH-Ser31 increased in all measured regions in HS rats. PrRP-receptor level decreased in the PVN in response to HS. Means ± SEM, Student’s *t* test, p*** < 0.001, p* < 0.05 vs. NS group, *n* = 4. *Ab* antibody, *AV3V* anteroventral third ventricular area, *AVPe* anteroventral preoptic nucleus, *MnPO* median preoptic nucleus, *OT* optic tract, *PrRP-R* PrRP-receptor, *pTH-Ser31* phosphorylated TH at Ser31, *PVN* hypothalamic paraventricular nucleus, *m* magnocellular and *p* parvocellular parts of the PVN, *SON* supraoptic nucleus, *VMPO* ventromedial preoptic nucleus, *3V* third ventricle

To better understand the molecular mechanisms of adaptation to chronic hypernatremia, we performed further investigations in the HS model. In harmony with the mRNA results, both TH (*p* < 0.05) and NUCB2 (*p* < 0.05) (43 kDa product) protein levels (measured by Western blot) were heightened by chronic HS intake in the cVLM (Fig. 6a). No alterations were measured in the amounts of these proteins in the cDMM (Fig. 6a). PrRP isoforms (PrRP31: 3.6 KDa, PrRP20: 2.6 KDa) were too small for western blot analysis. Therefore, the number of the PrRP-immunopositive cells was determined to evaluate expressional changes. The number of the PrRP-immunopositive cells was higher in the HS compared to NS group (*p* < 0.05) in the cVLM, in agreement with the mRNA measurements. However, in the cDMM, where we found reduced PrRP mRNA levels in the HS group, the number of the PrRP-immunopositive cells was maintained (Table 3).

**Table 3:** Prolactin-releasing peptide immunoreactivity in the different brain areas after high salt intake

	Cell number/section		Fiber density (μm^2^/100 μm^2^)			*n*
	cVLM	cDMM	AV3V	SON	PVN	
NS	6.3 ± 0.8	52.3 ± 3.7	1.2 ± 0.2	1.0 ± 0.1	4.2 ± 0.2	4
HS	10.8* ± 1.4	52.5 ± 5.4	3.0* ± 0.2	1.4* ± 0.1	10.2* ± 1.6	4

*AV3V* periventricular anteroventral third ventricle region, *cDMM* caudal dorsomedial, *cVLM* caudal ventrolateral medulla oblongata, *PVN* hypothalamic paraventricular nucleus, *SON* supraoptic nucleus. Means ± SEM, Student’s *t* tests, p* < 0.05 vs. NS controls

PrRP and TH innervations were analyzed in the main osmoregulatory centers: the AV3V, PVN and SON. The PrRP fiber network was the richest in the PVN. More than two-thirds of the analyzed varicosities contained both TH and PrRP immunoreactivity in the AV3V and the PVN, and almost all were double labeled in the SON in control rats (Fig. 6b, c). Thus, the majority of the PrRP-positive fibers originated from the A1 or A2 cell groups. HS intake enhanced the density of the PrRP-positive fibers in all of the investigated regions (*p* < 0.05), (Table 3, Fig. 6d, e). Within the PVN, this was more obvious in the medial parvocellular region and less in the magnocellular parts of the nucleus, where the PrRP fiber network was poor (Fig. 6e). Several neurons expressed PrRP-R in the AV3V (mainly in the MnPO and in the anteroventral periventricular nucleus) and the medial parvocellular PVN. Neurons in the magnocellular PVN and SON failed to express PrRP-R (Fig. 6f). Chronic hypernatremia downregulated the amount of the PrRP-Rs (measured by Western blot) in the PVN (*p* < 0.05). The decrease in the AV3V did not reach the level of significance (Fig. 6g). Parallel with the increased PrRP innervation, TH phosphorylation at Ser31 (TH-Ser31) was upregulated in all of the HS samples (AV3V, SON: *p* < 0.05, PVN: *p* < 0.001) indicating a stimulated catecholamine release (Fig. 6g).

## Discussion

Chronic hypernatremia triggers central reactions affecting the neuroendocrine, autonomous and behavioral circuits. NE and PrRP containing medullary axons target critical hypothalamic centers organizing these reactions and probably exert an enhanced signaling during chronic hypervolemic hypernatremia. Cooperation of NE and PrRP under this condition may represent a basis for the ongoing adaptation mechanisms. Contribution of nesfatin acting by autocrine/paracrine manner within the cVLM and cDMM is also suggested.

High salt solution intake for 6 days resulted in the development of typical, somatic signs of chronic stress due to the chronic, mild elevation of the resting plasma corticosterone level in adult rats. In contrast, genetically AVP deficient adult DI rats exhibited no somatic signs of chronic stress and normal basal plasma ACTH and corticosterone concentrations. The difference between the two models may explain the above findings. DI causes euvolemic hypernatremia, as an outcome of water loss through the kidneys, without high blood pressure (Sun 2006). In contrast, sodium overconsumption causes hypervolemic hypernatremia, elevated plasma AVP concentration and blood pressure (Choe et al. 2015). The plasma osmolality in the DI group was significantly lower than in the HS group. The mean corrected food intake of DI rats was normal. HS intake, however, caused dehydration-induced anorexia (Boyle et al. 2012). During the embryonic development, several alterations take place in the expression of different genes and neuronal pathways in DI rats to compensate for the lack of AVP (Scholer and Sladek 1981; Bundzikova et al. 2008; Pouzet et al. 2001; Yang and Coote 2007). Plasticity in response to challenges in adulthood is much more limited. All in all, HS intake represented a higher degree of homeostatic threat for the cardiovascular and osmotic control as well as the regulation of the energy balance. This definitely produced a higher level of stress in animals challenged by HS intake compared to DI rats.

Severe functional impairment of the HPA axis was displayed only when hypernatremia evoked serious chronic stress: the ACTH and corticosterone responses to acute restraint were absent in HS-R animals, while they were almost intact in the DI-R group. These hormonal responses to acute restraint greatly depend on the ascending medullary NE transmission towards the PVN (Gaillet et al. 1991; Pacak et al. 1995; Flak et al. 2014). Indeed, the acute restraint upregulated the expression of TH in the A1 cell group in the non-hypernatremic rats in both models (WT-R, NS-R). It seemed, however, that the type of hypernatremia affected the basal level of TH in the A1 cell group differentially (normal in DI and high in HS rats), and the level of TH determined the responsiveness of the HPA axis for the acute restraint (normal in DI-R and blunted in HS-R rats). The correlation data between the TH and ACTH/corticosterone measurements confirmed this idea. Thus, a ceiling effect in the TH (the rate limiting enzyme for NE synthesis) expression may have contributed to the impaired HPA axis responsiveness.

The exhaustion of TH capacity in the A1 cell group in chronic HS intake occurs probably for complex reasons. Chronic HS intake challenges strongly the osmotic and cardiovascular regulations and NE of A1 origin is crucial for these functions (Day 1989; Fernandez-Galaz et al. 1994; Tanaka et al. 1997; Pedrino et al. 2008). It probably also plays an important role in the chronic activation of the HPA axis during hypernatremia, since the medullary catecholamine cell groups mediate HPA axis responses for stimuli that signal homeostatic perturbations (Ulrich-Lai and Herman 2009; Gaillet et al. 1991; Pacak et al. 1995; Ritter et al. 2003; Bienkowski and Rinaman 2008; Khan et al. 2011).

According to our data, PrRP and nesfatin may assist within certain limits when the need for NE is high. Moreover, the regulation of the TH, PrRP and nesfatin genes is coordinated. Both were upregulated by restraint and by chronic hypernatremia in the cVLM in both models. The acute restraint evoked PrRP response was in association with the TH capacity and the overload of the stress axis. Thus, it was maintained in the DI, but not the HS loaded rats. The nesfatin response to restraint was blunted by prior chronic hypernatremia in both models. Coordinated regulation of the genes coding TH, PrRP and nesfatin within the same cell can happen in several ways. Glucocorticoids exert central feedback in stress by regulating gene expression. Glucocorticoid signaling is altered in the A1 neurons by chronic stress (Kitayama et al. 1989), which may affect the expression of TH, PrRP and nesfatin. The expression of these genes may also be affected by neural inputs modulating the excitability of cells. Such inputs may arrive primarily from the cDMM, forwarding essential information from the peripheral baro- and osmoreceptors (Chan et al. 1995), and from the hypothalamus, exerting a descending control (Toth et al. 1999; Bourque 2008). Moreover, local regulatory short loops may be important in neuromodulation. Nesfatin is a good candidate acting by an autocrine/paracrine way (Foo et al. 2008). In vitro effects of nesfatin on the excitability of cDMM and PVN neurons have already been demonstrated (Price et al. 2008; Mimee et al. 2012).

During habituation to chronic salt loading, restraint and inflammatory stress upregulation of AVP was observed in the CRH neurons of the parvocellular PVN (Harbuz et al. 1992; Ma and Lightman 1998; Amaya et al. 2001; Toth et al. 2008). AVP may contribute to ACTH (and corticosterone) secretion acting on the anterior lobe of the pituitary via V1b receptors in synergism with CRH (Antoni 1993). Actually, the role of parvocellular AVP was emphasized in the development of the blunted ACTH response to acute stress (restraint) in Sprague-Dawley rats with chronic osmotic stimulation (water deprivation) (Grinevich et al. 2001). However, the ACTH response was just marginally impaired in DI rats with no AVP, compared to the HS intake rats. This controversy may also be due to the limitation of the knock-out model. Nevertheless, upregulation of PrRP in the NE neurons takes place during adaptation to chronic restraint in the medulla (Toth et al. 2008). By demonstrating the upregulation of the coexpressed neurotransmitters in the cVLM in chronic hypernatremia, we showed that this phenomenon is part of a general mechanism in habituation to chronic stressful stimuli.

The adaptation of the “healthy organism” to chronic hypernatremia has further been investigated in the HS model with aggressive alterations in the measured parameters and lacking the compensatory mechanisms that had been developed in the AVP-deficient rats throughout the ontogenesis.

TH, PrRP and NUCB2 protein levels were all elevated in the cVLM in the HS group compared to the NS group. This was in harmony with the mRNA data pointing to a balanced reaction to an increased demand. In the cDMM, discrepancies were found between the mRNA and protein data, pointing to an increased demand for PrRP and nesfatin that disturbed the dynamics between the synthesis and transport/release (Sterrenburg et al. 2011).

The increased NE and PrRP turnover during high salt loading was confirmed by the examination of the hypothalamic terminal fields. The elevated pTH-Ser31 levels pointed to an ongoing NE release (Dunkley et al. 2004; Haycock 1993; Daubner et al. 2011; Ong et al. 2012) in the SON, PVN and the AV3V in HS rats. Undoubtedly, since NE innervation of these areas arises almost exclusively from the A1 and A2 cell groups (AV3V: ~ 100%, PVN: 94%, SON: 89%), NE has been released primarily from the axons of the A1 and A2 cells (Sawchenko and Swanson 1981). The A1 and A2 neurons are practically identical to PrRP neurons (Chen et al. 1999), and a very high percentage of the PrRP-positive fibers was of A1 or A2 origin in the AV3V, PVN and SON. Together with the heightened density of PrRP-positive fibers found in the investigated hypothalamic regions in the HS group, these results provide convincing evidence that the sustained NE release is linked with PrRP corelease.

Regarding the known cooperation between NE and PrRP in the AVP and ACTH release (Maruyama et al. 2001; Uchida et al. 2010), as well as in habituation to chronic stress (Toth et al. 2008), we assume that PrRP acts synergistically with NE in the different responses to chronic hypernatremia. Coordinated or independent PrRP release, however, from the non-NE PrRP cells located in the dorsomedial nucleus cannot be excluded.

Several central behavioral, autonomic and hormonal reactions are triggered by the increased plasma sodium level. These include the generation of thirst, reducing sodium appetite, increasing renal sodium excretion and water retention, elevation of blood pressure and parallel activation of renal sympatho-inhibition (Bourque 2008; Dos Santos Moreira et al. 2017). Noradrenergic inputs to the SON, PVN and the AV3V from the A1 and A2 cell groups are essential to trigger these reactions (Fernandez-Galaz et al. 1994; Pedrino et al. 2005, 2008, 2012; Dos Santos Moreira et al. 2017). Based on our results, the central role of PrRP in all these functions is therefore emerging.

In the parvocellular PVN, not only the density of the PrRP fibers was enhanced, but the receptors were also downregulated, further confirming the augmentation of PrRP release in HS rats. This highlights the putative role of PrRP in the control of the dehydration-induced anorexia (in addition to the already discussed regulation of the HPA axis). Both food intake and bodyweight of HS rats were reduced. PrRP acts as an anorexigen, which is mediated by CRH neurons in the parvocellular PVN (Lawrence et al. 2004). Negative energy balance (e.g. food restriction, lactation) therefore downregulates the expression of PrRP (Lawrence et al. 2000). In dehydration-induced anorexia, however, central mechanisms might be turned on to reduce food intake, which is a way to compensate the excessive salt intake (Boyle et al. 2012). Thus, expression of PrRP is expected to be increased, which is in harmony with our results.

In the magnocellular PVN and SON, PrRP innervation was weak, but inducible by HS intake. PrRP-Rs were present barely. Still, a single dose of icv. PrRP is able to stimulate the release of magnocellular oxytocin and AVP (Maruyama et al. 1999; Uchida et al. 2010). As oxytocin regulates natriuresis and AVP regulates water conservation by the kidneys as well as increases blood pressure, both hormones substantially control the hypernatremia-evoked responses. Although direct action of PrRP mainly on oxytocin neurons is possible (Maruyama et al. 1999), our data indicate that PrRP stimulates hormone release from the magnocellular neurons via an indirect pathway.

We recognized the AV3V as a new site for PrRP’s action. The AV3V exhibited a significant amount of PrRP-Rs and a great network of PrRP-positive fibers. In addition, PrRP innervation of the MnPO was enhanced by HS intake. The AV3V supervises the release of AVP and oxytocin (Honda et al. 1990; Yamaguchi and Yamada 2008; Tanaka et al. 1997) and plays a decisive role in the processing of autonomic responses elicited by HS intake (Pedrino et al. 2005; Bourque 2008). Therefore, it may serve as a relay center toward the magnocellular system. The effect of PrRP on the blood pressure (Samson et al. 2000; Yamada et al. 2009) was shown to be greatly mediated by neuropeptide FF2 receptors in the parvocellular PVN (Ma et al. 2009). However, according to our data the AV3V may also mediate this action via PrRP-Rs. Indeed, a polymorphism of the PrRP-R, the only cognate receptor of PrRP, has been associated with lower blood pressure in humans (Bhattacharyya et al. 2003).

In summary, we have found that the level of stress caused by chronic hypernatremia depended on the type of hypernatremia, affected the responsiveness to acute restraint differentially, and that the sensitivity of the HPA axis to acute restraint was reflected by the expression pattern in the cVLM. An interaction of NE and PrRP of medullary origin in the hypothalamus (PVN, SON, AV3V) in response to chronic hypernatremia was revealed and the AV3V was identified as a new site for PrRP’s action.
